# Diseases of the Throat, Nose and Ear

**Published:** 1915-12

**Authors:** 


**Diseases of the Throat, Nose and Ear.** By WILLIAM H. KELSON. Pp. xv. 270. Illustrated. London: Henry Frowde & Hodder & Stoughton. 1915.

The book has been written for general practitioners and senior students, and " so full details are given of such proceedings as the doctor himself usually undertakes, *i.e.* the removal of cerumen, while brief reference only is made to those which he generally passes on to the specialist." We do not find that these details are sufficiently full, whereas the exceedingly sketchy character of the descriptions of many of the diseases of the regions comprised in the scope of the book are inadequate.

The illustrations are numerous, and many of them excellent.

It must be presumed that the work reflects the present-day teaching of the staff of the London Throat Hospital, Golden Square.

